# Temporal Stability of Vegetation Cover across the Loess Plateau Based on GIMMS during 1982–2013

**DOI:** 10.3390/s21010315

**Published:** 2021-01-05

**Authors:** Chunyan Zhang, Shan Guo, Yanning Guan, Danlu Cai, Xiaolin Bian

**Affiliations:** 1 Aerospace Information Research Institute, Chinese Academy of Sciences, Beijing 100101, China; zhangcy@radi.ac.cn (C.Z.); guoshan@aircas.ac.cn (S.G.); caidl@radi.ac.cn (D.C.); bianxl@radi.ac.cn (X.B.); 2 Laboratory of Target Microwave Properties, Deqing Academy of Satellite Applications, Deqing 313000, China

**Keywords:** vegetation, persistent time, Global Inventory Modeling and Mapping Studies (GIMMS), coefficient of variation

## Abstract

The Loess Plateau, covering approximately 640,000 km^2^, has experienced the most severe soil erosion in the world. A greening tendency has been noticed since implementing the Grain to Green Program (GTGP), which may prevent further soil erosion. Therefore, understanding the underpinning basis of greening stability and persistence is important for sustainable improvement. Global Inventory Modeling and Mapping Studies (GIMMS) normalized difference vegetation index (NDVI) datasets for 1982–2013 were used to investigate the temporal stability and persistent time (PT) of vegetation over the Loess Plateau, utilizing the coefficient of variation (CV) and the estimation of tendencies of vegetation greening starting from the selected reference conditions. Two periods from 1982 to 1999 (as the reference period) and 2000 to 2013 were selected by considering the GTGP since 1999. The results indicate that: (1) A significant increase in vegetation cover occurred in the low NDVI area (NDVI < 0.3), with a high fluctuation from 2000 to 2013 compared with the reference period. Moreover, the fluctuation in vegetation is more related to precipitation variation since 1999. (2) Most areas recovered in the greening trend of the first period starting in 2009, occurring in 28.7% (2628 of 9148) of the total area. (3) The revegetated areas have a low PT and a high $CV_{vi}$, that is, the revegetated areas need a long time to recover from disturbances. Therefore, we identify the sensitive areas with PT = 4; further management needs to be implemented for sustainable development in these areas. These results provide a method to quantify the stability and persistence of the complex interactions between vegetation greenness and environmental changes, particularly in fragile areas.

## 1. Introduction

Many restoration projects have been conducted to recover degraded ecosystems around the world [1]; the social and environmental problems related to large-scale revegetation have been the subject of considerable debate [2]. The Grain to Green Program (GTGP) is the largest ecological restoration program in water-limited areas. It has been implemented across the Loess Plateau of China since 1999 [3]. This program aimed to increase the vegetation coverage by converting sloping land (located in hilly slopes) or barren land to forests or grasslands [4]. The program has induced a significant increase in vegetation cover in the context of climate change [3], and its effects on water–energy dynamics and climate have varied among different areas due to different methods of implementation and vegetation functioning [5,6].

The arid and semi-arid Loess Plateau of China has experienced the most severe soil erosion in the world [7,8], and water scarcity constrains the growth of vegetation [9]. With restoration project implementation, the land cover has changed significantly on the Loess Plateau, and vegetation cover has increased significantly between 1999 to 2013 [3]. As proxies of vegetation health, remote sensed vegetation indexes, for example, normalized-difference vegetation index (NDVI), enhanced vegetation index (EVI), and leaf area index (LAI), are used to examine vegetation changes across the Loess Plateau [10,11,12,13]. The vegetation cover decreased during 1982–1999 [10], while the GTCP induced a 25% increase in vegetation cover during 2000–2010 [11]. However, problems such as increasing drought and excessive soil erosion have been observed in many places on the Loess Plateau [9,14]. The effects of extreme climate events, which augment climate variabilities and also impose increased stress on environments, have been reported globally [15,16]. Extreme precipitation is especially severe and heterogeneous across the Loess Plateau [17,18]. In the context of climate change, the vegetation greening process, vegetation cover stability, and ability of vegetation to recover from perturbations, are important for sustaining the functions, structures, and patterns of the local vegetation ecosystem [19].

Plant growth and maximum plant coverage are limited by low water availability across the Loess Plateau [20]. The vegetation resilience time (the ability of vegetation to tolerate disturbances and to recover their initial state [21]) depends on plant types and attributions [22], and the frequency and magnitude of the environmental perturbations [21,22], including drought, wildfires, high winds, landslides, floods, avalanches, and extreme climate events. The vegetation persistence, which relates to the ability of vegetation to recover from climatic or anthropogenic disturbances, can provide realistic and useful parameters of vegetation resilience [23]. If the vegetation recovery time from environmental disturbances can be estimated and used as a reference in further revegetation planning, the restoration will be more successful and effective.

Both environmental and anthropogenic changes affect the spatial and temporal dynamics of vegetation [24,25]. Vegetation dynamics and the relationship with climate change have been extensively reported in previous studies [16,25,26,27,28,29], and precipitation and human activities have significant positive effects on NDVI trends. Recently, vegetation greening and its effects on the ecosystem of the Loess Plateau have been reported by a few studies [3,30,31,32,33,34,35,36,37,38]. Vegetation greening induced both net primary productivity and evapotranspiration to increase, in turn causing a significant decrease in the ratio of river runoff to annual precipitation. However, how fast the vegetation can recover, that is, the persistence of vegetation cover is still unknow since 1999 when the GTGP was implemented on the Loess Plateau. Thus, in this study, we focused on the spatiotemporal aspects of the observed vegetation greening, and locating the areas requiring further attention. Therefore, the aims of this study were (1) to assess the temporal stability, measured by coefficient of variation (CV); (2) to investigate the persistence of vegetation greening using the revised first-passage statistic method based on NDVI datasets developed by the Global Inventory Modeling and Mapping Studied (GIMMS); and (3) to examine the relationship between precipitation and vegetation due to the water-limitation across the Loess Plateau. By mapping the temporal stability and vegetation persistence, the vegetation greening process at a given location can be determined, and the highly sensitive areas can be identified for further revegetation planning.

## 2. Materials and Methods

### 2.1. Study Area

**Figure 1:**
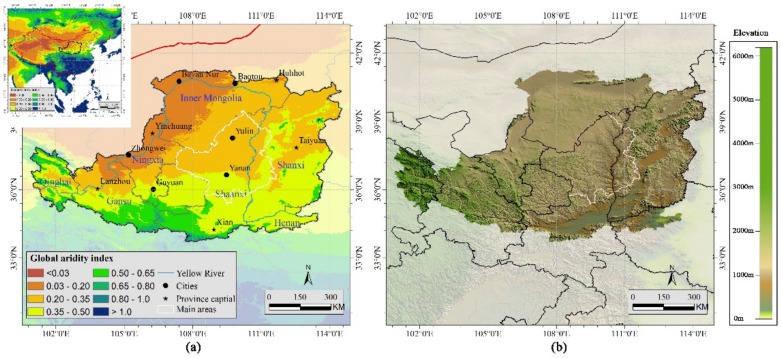
The study area with (**a**) aridity index, and (**b**) elevation.

The Loess Plateau, spanning approximately 640,000 km^2^, is in the arid and semi-arid region of northwestern China (Figure 1), and is known for its severe soil erosion and water loss induced by unregulated land use and predatory resource utilization [30,39,40]. The Loess Plateau covers five provinces and two autonomous regions, including Shanxi, Shaanxi, Gansu, Qinghai, Henan, Ningxia, and Inner Mongolia. The aridity index, which is calculated as the ratio of mean annual precipitation to mean annual potential evapotranspiration [41,42], of the Loess Plateau gradually increases from the northwest to southeast. The climate is characterized by the East Asian Summer Monsoon, with high intensity summer rainstorms [43], and the mean annual precipitation increases from 200 mm in the northwest to 700 mm in the southeast, with the mean temperature ranging 8–14 °C [20]. To restore the degraded ecological environment and promote soil stability, a diverse range of soil conservation approaches (including terracing and construction of check-dams) and ecological approaches (GTGP) have been implemented in the Loess Plateau [44]. The main areas that conducted GTGP projects are in the northern part of Shaanxi province and the western part of Shanxi province, as shown in Figure 1 [3].

### 2.2. Remote Sensing Vegetation Datasets

NDVI, ranging from −1.0 to 1.0, quantifies the photosynthetic activity of vegetation by measuring the difference between the near infrared and red reflectance divided by the sum of the two [45]. Only positive values correspond to vegetated areas; the higher the value, the greater the target chlorophyll content [46]. The NDVI time series that covers 1982–2013 was extracted from the Advanced Very High Resolution Radiometer (AVHRR) NDVI 3rd generation (NDVI3g, https://ecocast.arc.nasa.gov/data/pub/gimms/3g.v0/), which was developed by the GIMMS group, for the determination of vegetation stability across the Loess Plateau. The GIMMS NDVI3g data are provided every 15 days at a spatial resolution of 8 km as a gridded product in the geographic lat/lon projection based on WGS-84 datum. The data set was derived from imagery obtained from the AVHRR instrument onboard the NOAA satellite series 7, 9, 11, 14, 16, and 17. This dataset has been corrected for calibration, view geometry, volcanic aerosols, and other effects not related to vegetation change [47], and shows better performance at high latitudes and has a better calibration capability than earlier versions owing to the use of Sea-Viewing Wide-Field-of-View Sensor (SeaWifs) data [48]. Compared with other NDVI products, this dataset elicited good linearity with Systeme Probatoire d’Observation de la Terre (SPOT) NDVI [49] and MODIS NDVI [26]. It has been used to analyze the global trends in seasonality [50] and quantify the effect of the human footprint on NDVI trends [51]. The maximum value compositing procedure was used to minimize the effects of cloud contamination, varying solar zenith angles, and surface topography [52]. The annual mean of NDVI is calculated based on the monthly maximum NDVI.

### 2.3. Land Cover Data

To ensure the correspondence of the time-series land cover data, the MODIS land cover type product (MCD12Q1) was collected for this study. MCD12Q1 supplies annual global land cover maps at 500 m spatial resolution in geographic lat/long projection for 2001–present. The product contains five land cover classification schemes, including the International Geosphere-Biosphere Programme (IGBP), University of Maryland (UMD), leaf area index (LAI), BIOME-biogeochemical cycles (BGC), and plant functional types (PFT). The IGBP classification system was used in this study. This dataset was downloaded from the United States Geological Survey (USGS) website (https://lpdaac.usgs.gov/products/mcd12q1v006). The original classes were reclassified into six classes for this study (Table 1). To improve the stability of the MODIS land cover product, a hidden Markov model framework was developed to minimize the amount of spurious land cover change [53]. However, it still needs to be cautiously used to determine the land cover change. The land cover datasets for 2001 and 2013 were used to analyze the changes in land cover types. New croplands, new forests, new grasslands, and new shrublands were extracted as revegetation areas.

### 2.4. Climate Data

Precipitation is the main source of soil water on the Loess Plateau. Effective precipitation and soil water storage are the main water resources for plant growth. Therefore, the effects of precipitation on vegetation were investigated. Precipitation, derived from the China Meteorological Forcing Dataset (http://westdc.westgis.ac.cn/data/7a35329c-c53f-4267-aa07-e0037d913a21), was used in this study. This dataset was developed by Data Assimilation and Modeling Center for Tibetan Multi-Spheres, Institute of Tibetan Plateau Research, Chinese Academy of Sciences [54]. The spatial resolution is 0.1° and its temporal resolution is three-hourly, monthly, and yearly with the WGS-84 geographic coordinate system. The annual precipitation datasets were used to investigate precipitation variations. All datasets were resampled using the nearest neighbor method and converted into the WGS-84 geographic coordinate system with WGS-1984 datum in ArcGIS software to obtain a comparable resolution of 0.08° for the GIMMS NDVI dataset. In other words, all the data were resampled to the same resolution of 0.08°.

### 2.5. Persistence Analysis

To better understand the vegetation greening processes across the Loess Plateau, the persistence of vegetation greening was assessed using the first-passage statistic [55,56]. This method has been used to investigate the persistence of vegetation in southern Africa, Italian Mediterranean, and other Italian regions [23,55,56]. In the analysis, the reference period needs to be selected to produce the linear regression slopes surface ($s\left(x,y,t_{ref}\right)$), which represents the slope of a given pixel $\left(x,y\right)$ over the reference period $t_{ref}$, as a reference, and NDVI values are added to create a new slope surface ($s\left(x,y,t_{i}\right)$) with a yearly time step. Then, the persistence maps of each year are constructed by comparing $s\left(x,y,t_{i}\right)$ and $s\left(x,y,t_{ref}\right)$. For pixels $\left(x,y\right)$, the persistence value $P\left(x,y,t_{i}\right)$ is assigned a value of 1 when $s\left(x,y,t_{i}\right)$ > $s\left(x,y,t_{ref}\right)$, otherwise a value of −1 is assigned. 

However, if vegetation is stable or has a slightly increase compared with the reference period, the new time slope will be lower than that of the reference period. Therefore, we revise it using the vegetation trend increment, Δndvi, and we computed $\Delta ndvi\left(x,y,t_{i}\right)$ using the following formula according to trigonometric tangent function:

(1) $$\Delta ndvi\left(x,y,t_{i}\right)\;=\;s\left(x,y,t_{i}\right)\times \left(t_{i}-t_{0}\right),$$

where $t_{i}$ and $t_{0}$ represent the end year and the start year, respectively. Δndvi based on the NDVI trend removes the effects of noise and NDVI fluctuation, but is sensitive to slight changes. Therefore, the persistence value $P\left(x,y,t_{i}\right)$ is assigned according to the following rules:

(2) $$P\left(x,y,t_{i}\right)\;=\;\left\{\begin{array}{c} 1\;\;\;\;if\;\Delta ndvi\left(x,y,t_{i}\right)\;\geq \;\Delta ndvi\left(x,y,t_{0}\right)\\ \;0\;\;\;\;if\;\Delta ndvi\left(x,y,t_{i}\right)\;<\;\Delta ndvi\left(x,y,t_{0}\right)\; \end{array}\right.,\;t_{i}>t_{0}.$$

Summing all the persistence maps over the observed period, a cumulative persistent time (PT) map is obtained. For each pixel, the PT value reveals the number of years in which vegetation continues the increasing trend. A high PT value indicates a long PT of the NDVI greening trend. The higher the PT value, the shorter the recovery time. From the PT map, vegetation greening processes across the Loess Plateau can be identified. The period from 1982 to 1999 was selected as the reference period due to the implementation of GTGP from 1999. 

### 2.6. Temporal Stability

Ecosystems that are highly sensitive to disturbances and slowly return to their equilibrium state will have a larger variability compared to ecosystems that are insensitive to perturbations and rapidly return to equilibrium [21]. Thus, the temporal stability was measured by the CV as the ratio of standard deviation to the mean:

(3) $$CV=\frac{Std\left(DN_{i}\right)}{Mean\left(DN_{i}\right)},\;i\epsilon \left\{1,\ldots ,n\right\},$$

where $DN_{i}$ is the mean value of NDVI or annual precipitation in a particular year, and *Std* and *Mean* are the standard deviation and mean of years, respectively. The temporal stability of vegetation and precipitation were examined in two periods (1982–1999, and 2000–2013). The relationships between the temporal stability of vegetation and precipitation are determined to identify the effects of precipitation on vegetation.

## 3. Results

### 3.1. The Land Cover Changes

**Figure 2:**
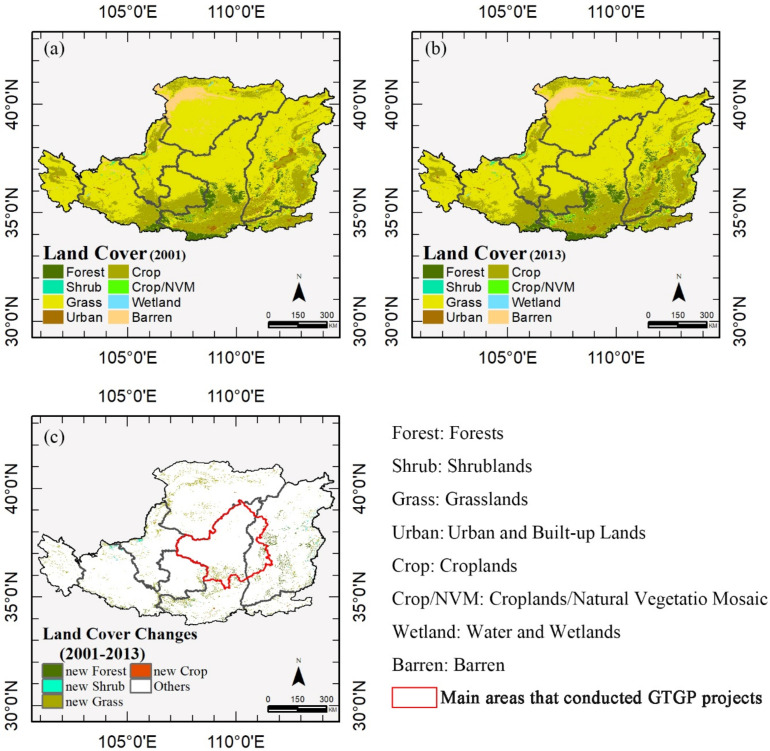
Land cover types in (**a**) 2001, (**b**) 2013, and (**c**) between 2001 and 2013 (only showing the new forests, shrublands, grasslands, and croplands). The red boundary indicates the outline of the main areas of the counties that implement the Grain to Green Program (GTGP).

In 2001, approximately 68.39% of the Loess Plateau was covered by grasslands, 21.07% by croplands, and 4.93% by forests (Figure 2a and Table 2). In 2013, grasslands were reduced to 64.53%, with croplands reduced to 24.23% and forests to 6% of the land (Figure 2b and Table 2). Thus, the decline in grasslands was partially compensated by an increase in croplands and forests. Conversions from croplands and grasslands to croplands/natural vegetation mosaic were also observed. Examination of the land cover distribution in 2001 and 2013 as shown in Table 2, revealed consistent regional patterns. Grasslands were prevalent in the northwestern areas, croplands were concentrated in the low valleys of the Loess Plateau, and forests covered the high mountainous areas. The land cover of the main area is composed of forests, croplands, and grasslands.

The most important land cover change was a decline in grasslands and an increase in forests and croplands. The increase in forests occurred in the eastern Loess Plateau (especially in the central part of Shanxi and Shaanxi province), and an increase in croplands was observed at Shanxi province. The new vegetation areas (Figure 2c), including new croplands, new forests, new grasslands, and new shrublands, were extracted as the revegetation areas. Only small revegetation areas were detected in the southern and eastern sections of the main areas.

### 3.2. Temporal Stability and Change Tendency of Vegetation

**Figure 3:**
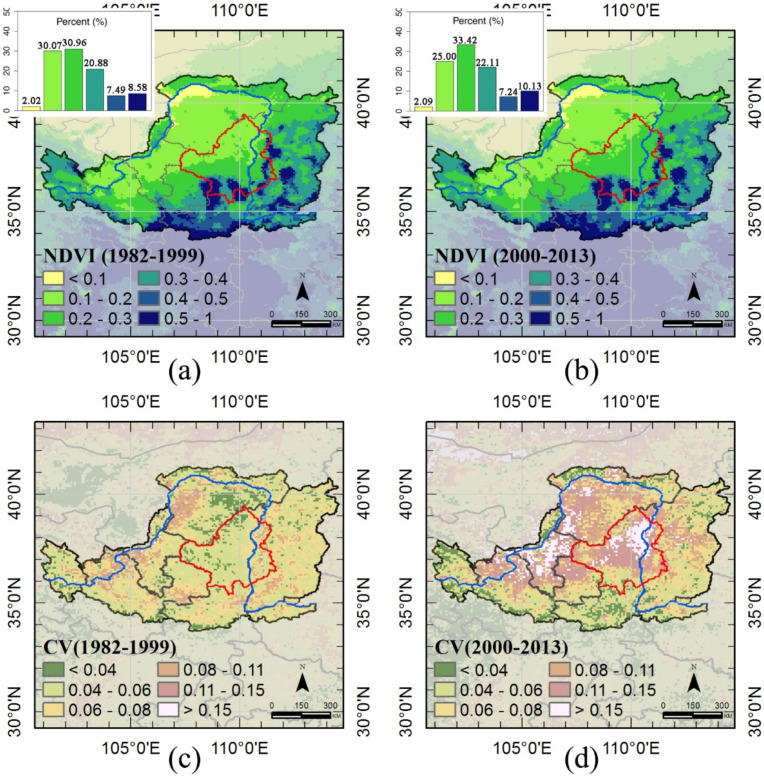
Spatial distribution of long-term averaged vegetation greenness and the coefficient of variation (CV) of vegetation greenness: (**a**,**c**) 1982–1999, (**b**,**d**) 2000–2013. The red boundary indicates the outline of the main areas of the counties that implemented the Grain to Green Program (GTGP). The blue line is the Yellow River. The smaller images in (**a**,**b**) shown the area percentages of each classes demonstrated in the large one.

The geographical distribution of long-term averaged NDVI for two periods (1982–1999 and 2000–2013) also show similar patterns (Figure 3). Our results suggest that the Loess Plateau is dominated by moderate density vegetation, with NDVI < 0.4. Low-density vegetation was mainly found in the arid northwestern region of the Loess Plateau, and high-density vegetation was observed in the relative wet southeastern area of the Loess Plateau. For comparison, NDVI is classified into six categories, higher NDVI groups have an increase in vegetation greenness, excluding areas with NDVI between 0.4 and 0.5. The most important NDVI change was a decline in low value group, $\mathrm{NDVI}\in \left(0.1\sim 0.2\right)$, and an increase in moderate and high vegetation density areas, $\mathrm{NDVI}\in \left(0.2\sim 0.4\right)\;\mathrm{and}\;\left(0.5\sim 1\right)$, respectively. That is, vegetation changes mainly occurred in areas with low NDVI value. In addition, the increase of vegetation greenness in the main areas was obvious.

As shown in Figure 3c,d, the vegetation greenness $\left(CV_{vi}\right)$ patterns for two periods differed across the Loess Plateau, showing high heterogeneity, particularly in the second period. Low $CV_{vi}$ was most prevalent throughout the Loess Plateau during the first period, but an increase of $CV_{vi}$ was mostly located in the main areas and in the Ningxia province during the second phase. Areas covered by croplands were more stable.

**Figure 4:**
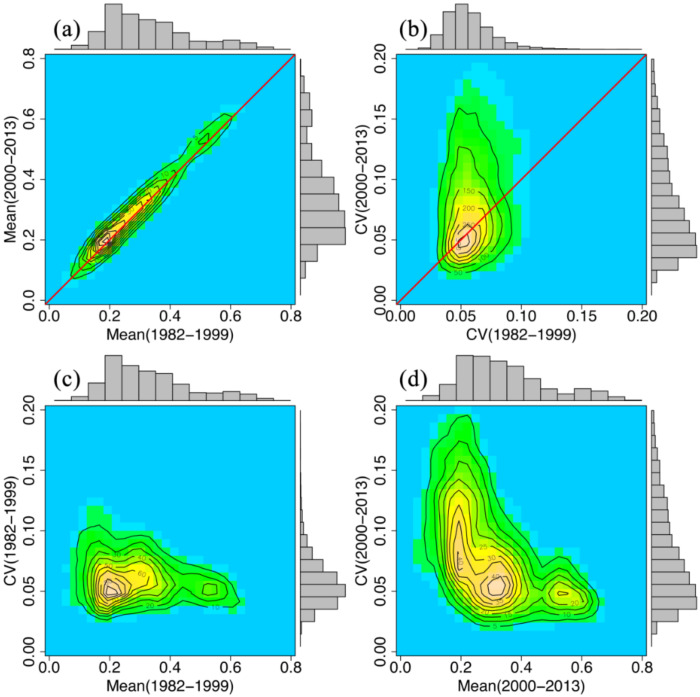
Interrelationship between long term averaged vegetation greenness and its coefficient of variation (CV) associated with frequency distributions are presented: (**a**) averaged normalized difference vegetation index (NDVI) (1982–1999 versus 2000–2013), (**b**) CV (1982–1999 versus 2000–2013), and NDVI versus CV in (**c**) 1982–1999, (**d**) 2000–2013, respectively.

To determine the relationship between vegetation greenness and its variations, frequency distributions were used. The vegetation greenness in the first period appeared to be strongly and positively correlated with the second period, but the greenness fluctuation in the first period was independent of that during the second period. The changes in $CV_{vi}$ were more apparent, and low values of $CV_{vi}$ in the first period changed to a wide range of $CV_{vi}$ values in the second period (Figure 4c,b). In other words, the low and moderate areas have varied over time since 1999.

**Figure 5:**
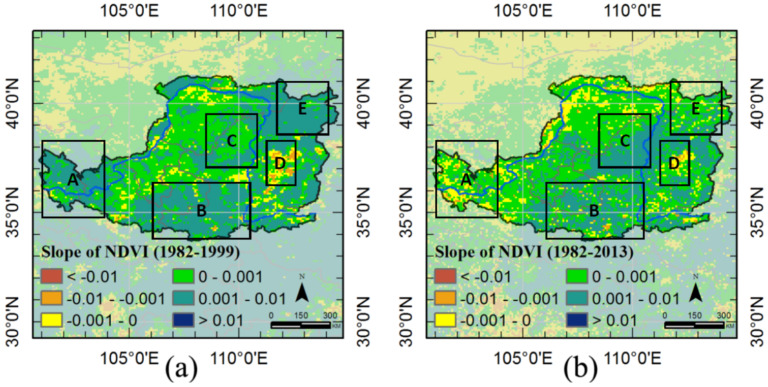
Vegetation greenness change tendency: (**a**) normalized difference vegetation index (NDVI) (1982–1999), (**b**) NDVI (1982–2013).

The tendencies of two periods (1892–1999 and 1982–2013) were investigated to reveal the changes in vegetation greenness. As shown in Figure 5, slopes > 0.001 occur in 53.03% of the Loess Plateau during 1982–1999, but only 35.52% during 1982–2013. Vegetation decay is found in the western and southern regions of the Loess Plateau (Frames A and B in Figure 5), northern Shanxi province (Frame E Figure 5), but vegetation greening is observed in the central part of the Loess Plateau and Shanxi province (Frames C and D in Figure 5).

### 3.3. Temporal Persistence of Vegetation

**Figure 6:**
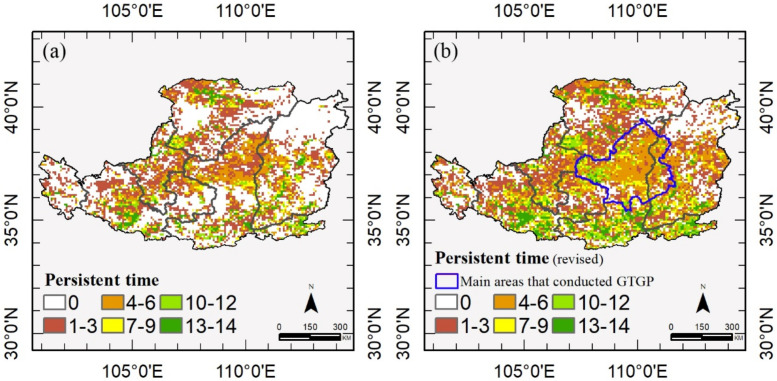
Persistent time of vegetation greening from 2000 to 2013 compared with the reference period (1982–1999) using (**a**) the first-passage statistic, and (**b**) the revised first-passage statistic. High the persistent time, short the recovery time.

The PT map of the Loess Plateau using 1982–1999 as the reference period is shown in Figure 6. High PT indicates vegetation greening and short recovery time, and zero indicates vegetation degraded or still not recovered from disturbances. The revised first-passage statistic is more sensitive than the first-passage statistic (Figure 6a), as demonstrated by more details (fewer zeros) in Figure 6b. High PT values occurred in the southern Loess Plateau, and the Hetao Plain, which were characterized by croplands (irrigated agriculture) or forests. Vegetation greening ceased in northeast of the Loess Plateau. Large clusters of low persistence of vegetation greening could clearly be observed within the main areas that participated in GTGP.

**Figure 7:**
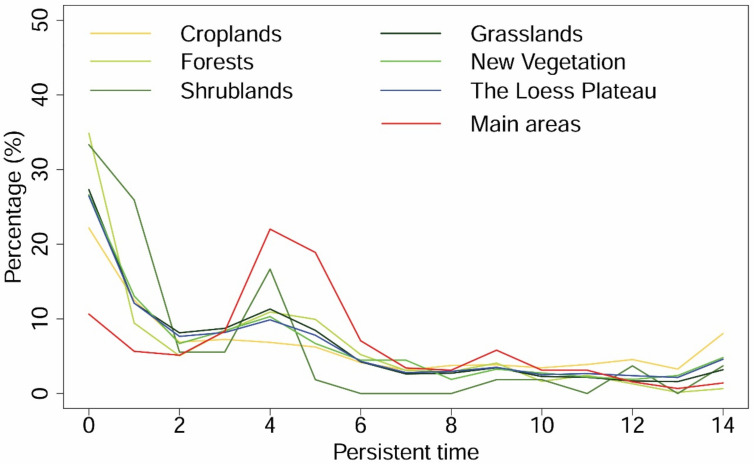
Persistent time of vegetation greening in different land cover types from 2000 to 2013 compared with the reference period (1982–1999). The regions of croplands, grasslands, forests, and shrublands are obtained from land cover of 2013 (MCD12Q1), and new vegetation includes the new regions of these four classes in 2013 compared with that of 2001.

Approximately one-tenth of the pixels (11.81%) had high PT over the Loess Plateau (PT > 10, the Loess Plateau in Figure 7). However, low PT dominated the Loess Plateau (54.41% pixels showed PT < 4), suggesting that decreasing trends were more common. It is notable that new vegetation areas had low frequency values (PT < 6, 71.82%, new vegetation in Figure 7), and the cropland-dominated areas were more persistent than other regions with a high PT (PT > 10, 19.75%, Figure 7).

**Figure 8:**
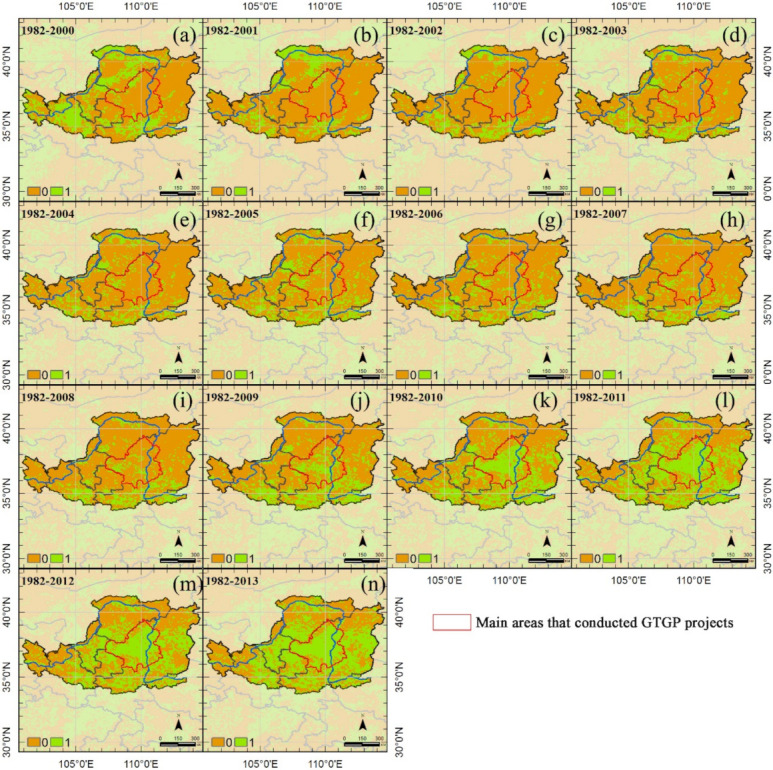
Persistence maps for the Loess Plateau over the period of 1982 to (**a**) 2000, (**b**) 2001, (**c**) 2002, (**d**) 2003, (**e**) 2004, (**f**) 2005, (**g**) 2006, (**h**) 2007, (**i**) 2008, (**j**) 2009, (**k**) 2010, (**l**) 2011, (**m**) 2012, and (**n**) 2013 compared with the reference period (1982–1999) using the revised first-passage statistic. Areas designated by 1 are those where the vegetation greening persisted, 0 indicates that vegetation greening ceased.

**Figure 9:**
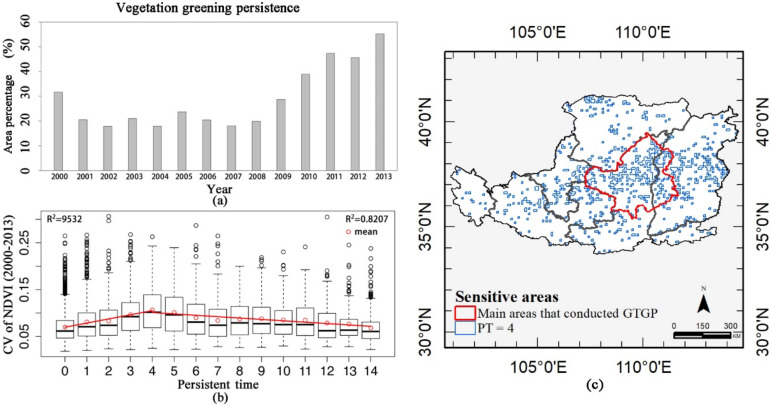
(**a**) Percentage of vegetation greening persistence from 2000 to 2013 using the revised first-passage statistic, (**b**) boxplot based linear regression statistic of persistent time (PT) of vegetation greening versus coefficient of variation (CV)of normalized difference vegetation index (NDVI), and (**c**) sensitive areas (areas with PT = 4).

For more details, the persistence map of each year from 2000 to 2013 is shown in Figure 8. One indicates vegetation greening persisted, zero indicates vegetation greening ceased. Vegetation in the western Loess Plateau sustained greening at the beginning of the restoration projects but fluctuated throughout the period. However, the main areas in which the GTGP was implemented show greening persistence from 2005, and most areas of vegetation greening have recovered since 2009 (green in Figure 8 and Figure 9a), occurring in 28.7% (2628 out of 9148) of the total areas. Additionally, the relationship between vegetation persistence and $CV_{vi}$ reveals that PT = 4 separates the relationship with $CV_{vi}$ (Figure 9b). $CV_{vi}$ was positively correlated with PT where PT < 4 but negatively correlated where PT > 4. Areas with PT = 4 had the highest $CV_{vi}$. Therefore, areas with PT = 4 are more sensitive to disturbances (Figure 9c). Most main areas are classified as sensitive areas.

### 3.4. Relationship Between Climate Fluctuation and Vegetation Fluctuation

**Figure 10:**
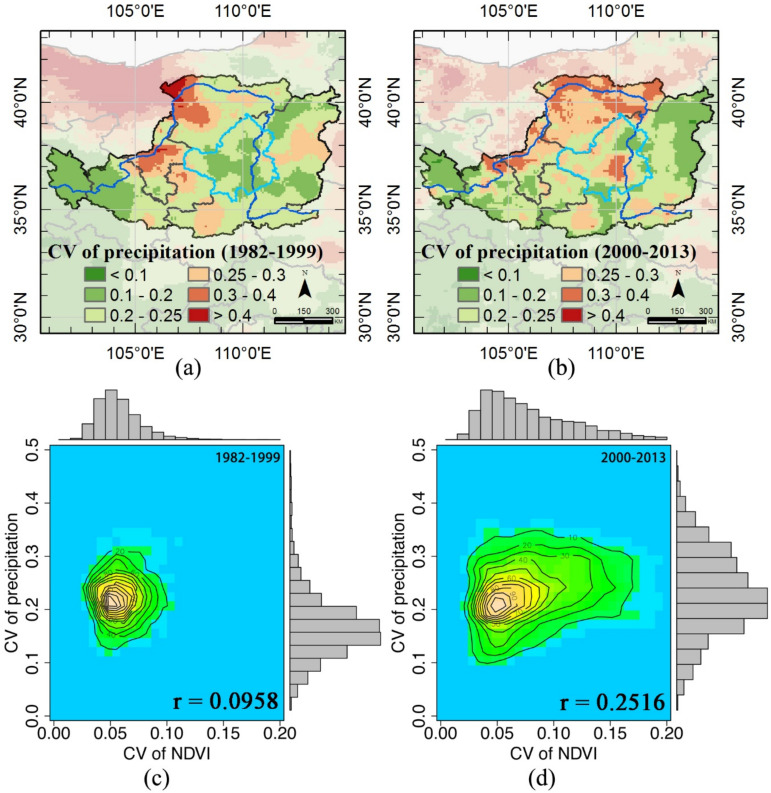
Coefficient of variation (CV) of precipitation: (**a**) CV (1982–1999), (**b**) CV (2000–2013). Frequency distributions between CV of normalized difference vegetation index (NDVI) and CV of precipitation: (**c**) 1982–1999, and (**d**) 2000–2013.

The CV of precipitation ($CV_{p}$) over two periods (1982–1992 and 2000–2013) was examined. High $CV_{p}$ values were observed in the northwestern Loess Plateau, and central Ningxia province during the first period (1982–1999); however, large areas showed high $CV_{p}$ values, including the main areas that conducted GTGP projects (Figure 10a,b). $CV_{vi}$ was independent of $CV_{p}$ during the first period, but was positively related to $CV_{p}$ during the second period with a coefficient, r = 0.2516 (Figure 10c,d).

## 4. Discussion

Recently, a vegetation greening trend was observed in some studies on the Loess Plateau [3,11]. A significant increase in vegetation greenness was also found in this research. The revegetation regions extracted from the MODIS land cover dataset are similar to the results of a previous study [11], but our revegetation areas are smaller. Their vegetation specifications were obtained from the Global Land Data Assimilation System (GLDAS), and we used the MODIS IGBP classification scheme. Different category schemes may induce different results. 

At the regional-scale, vegetation greening with high fluctuation dominated the observed inter-annual variability of NDVI. Our results indicate that the temporal stabilities of the areas with increasing vegetation fluctuated significantly. The temporal stability of vegetation is associated with an increasing drought frequency in the Loess Plateau [14]. In addition, the high $CV_{vi}$ of the northwestern Loess Plateau covered with grasslands is related to the fluctuation in precipitation, due to an enhanced close relationship with precipitation in the grassland [57,58]. Moreover, the main areas which conducted GTGP projects are more varied than other regions, and revegetation occurs on the rain-fed hilly regions of the Loess Plateau, where there is conflicting demand for vegetation water needs and actual water supply in water-limited areas [11]. 

In the long-time, the degradation of vegetation is more severe over the Loess Plateau, especially in the northeast areas. The PT of vegetation greening represents the recovery time of vegetation from disturbance. Our results indicated that low persistence values dominated the Loess Plateau. Indeed, previous studies concluded that vegetation in some mountains, including Liupan Mountains and Qinling Mountains, decreased [59]. However, croplands show high PT, because they have a relative high soil water content and soil water storage compared to grasslands and shrublands [60]. 

Surprisingly, the main areas have low PT, high $CV_{p}$ and high $CV_{vi}$, are more sensitive to both climatic and anthropogenic disturbance. Water is the primary limiting factor that constrains plant vegetation and economic development in this region [20], and the focus of the debate about revegetation across the Loess Plateau is the formation of a dry soil layer caused by the excessive introduction of exotic plant species along with high planting density [61,62]. A balance between water utilization by vegetation and soil water availability is the key to maintaining ecosystem health, particularly in the water-scarce Loess Plateau [3]. To avoid the degradation of revegetation, soil properties, vegetation types, stand ages [61,63] and plant cover density [20] need to be considered in future revegetation planning. Additionally, the current vegetation needs to be thinned in high density areas (to obtain optimal plant cover), and replaced with native and mixed species that utilize less water [3].

Climate change has had a more profound effect on vegetation since 1999. As shown in our results, the fluctuation in precipitation has a positive relationship with the variation in vegetation since 1999. Changes in precipitation slightly decreased during the first period, but experienced a dramatically reduction in precipitation during the second period [59], and high $CV_{p}$ during the second period, which is also related to more frequent extreme droughts during the second period [64]. Therefore, climate change has had more effects on vegetation growth since 1999, particularly in drier areas (corresponding to the low NDVI areas). 

Several large-scale ecological restoration projects, for example, the GTGP and the Three-North Shelter Forest Program (TNSFP), have been implemented, and their positive influences on improving vegetation greening and reducing land degradation and dust-storm intensity in northern China have been confirmed [65]. However, degradation continues to expand and intensify throughout the country [66]; for example, the death of *Populus* has been observed in Zhangbei County due to insect pests and plant regeneration. The sensitive areas suggested by Figure 9c correlate well with the severe rate of soil erosion, decreasing annual precipitation [18] and high precipitation variations, as shown in Figure 10b. Therefore, we need to pay more attention to the vegetation quality, although the vegetation cover has significantly increased. Moreover, suitable restoration schemes should be implemented to guide future vegetation restoration activities.

The accuracy of remote sensing data and climate data (e.g., inaccurate measures of NDVI in the Loess Plateau, which was not the focus of the current study) needs to be considered, with corrected data being integrated to improve its ability to identify changes in vegetation. In addition, the scale-dependence of the NDVI–climate relationship also needs to be considered, as shown by stationarity indexes at multi-scales for precipitation and temperature [67]; we believe, at the current scale, the relationship between NDVI and precipitation is reliable. The resolution of 0.08° cannot provide finer information, but is beneficial for its long time series. Moreover, the sensitivity areas identified by this study provide the key regions for further vegetation management.

## 5. Conclusions

In summary, this study is the first to investigate the process of vegetation greening, including temporal stability and vegetation persistence, across the Loess Plateau. Furthermore, we revised the persistence maps with the increment of NDVI based on the NDVI trends, which are more sensitive to slight changes than trends. As a result, we demonstrated the vegetation greening process in which vegetation recovers from environmental disturbance.

(1) We confirmed that the vegetation cover had significantly increased, but fluctuated since the restoration projects were implemented, particularly in main areas that conducted the GTGP projects. Low PT dominated the Loess Plateau, indicating a long recovery time for most areas and more vulnerability to disturbances than high PT areas. Furthermore, the recovery for most of vegetation greening started in 2009, with vegetation greening occurring in 28.7% of the total areas.

(2) Some sensitive areas with PT = 4 and high vegetation fluctuation were identified, and vegetation in these regions need more time to recover from environment disturbances and are more sensitive to climate change and human activities. The suitable vegetation types and management for a given location should be considered to better guide vegetation restoration activities in these areas.

(3) Precipitation was more varied both spatially and temporally, compared with the first period (1982–1999), and has a greater effect on vegetation greening during the second period (2000–2013), particularly in the drier northwestern regions.

These results improve our understanding of vegetation dynamics and the temporal stability and persistence of vegetation over the Loess Plateau. Incorporating climate change and anthropic changes could provide insights into the complex interactions between vegetation and environment changes. The underlying mechanism of the high PT but low fluctuations of vegetation areas will be helpful for further vegetation planning. Further management of vegetation plants needs to consider soil properties, vegetation types, stand ages, and plant cover density. Moreover, the current vegetation needs to be thinned in high density areas (to obtain optimal plant coverage), and replaced with native and mixed species that utilize less water.

## Figures and Tables

**Table 1:** Land cover classification of the International Geosphere–Biosphere Programme (IGBP) from MCD12Q1.

IGBP Classes	Reclassified Classes
Evergreen Needleleaf Forest	Forests
Evergreen Broadleaf Forests	
Deciduous Needleleaf Forests	
Deciduous Broadleaf Forests	
Mixed Forests	
Closed Shrublands	Shrublands
Open Shrublands	
Woody Savannas	Grasslands
Savannas	
Grasslands	
Urban and Built-up Lands	Urban and Built-up Lands
Croplands	Croplands
Cropland/Natural Vegetation Mosaic	Cropland/Natural Vegetation Mosaic
Permanent Wetlands	Water and wetlands
Water Bodies	
Permanent Snow and Ice	
Barren	Barren
Unclassified	Unclassified

IGBP classes can be found in the user guide to Collection 6 MODIS Land Cover.

**Table 2:** Land cover change from 2001 to 2013.

Land Cover Classes	Percentage (%)		Changes (%)
	Year 2001	Year 2013	
Forests	4.93	6.00	21.76
Shrublands	0.28	0.34	22.18
Grasslands	68.39	64.53	−5.64
Urban	1.82	1.91	4.80
Croplands	21.07	24.23	15.00
Croplands/Natural Vegetation Mosaic	0.16	0.32	93.30
Water and Wetlands	0.08	0.13	59.20
Barren	3.27	2.54	−22.21

## Data Availability

All data generated or appeared in this study are available upon request by contact with the corresponding author. Furthermore, the models and code used during the study cannot be shared at this time as the data also forms part of an ongoing study.
